# Don't Forget the Past! Understanding and Communicating Earth's History is Important for a Sustainable Future

**DOI:** 10.1002/gch2.70126

**Published:** 2026-06-30

**Authors:** Georg Feulner

**Affiliations:** ^1^ Earth System Analysis Potsdam Institute for Climate Impact Research Member of the Leibniz Association Potsdam Germany

**Keywords:** biodiversity, climate change, credibility, earth system science, ecological crisis, environmental crisis, environmental ethics, paleoclimatology, sustainability

## Abstract

The concept of sustainability has been shaped by the history of environmental problems and ecological crises, and the scientific value of studying the past has long been recognized. However, the relevance of studying Earth's past in the context of the sustainability debate goes beyond testing Earth‐system models used for future climate projections and looking for past analogues of future climate states or modern biodiversity loss. The past is also important for communicating the climate and ecological crisis in terms of enhancing scientific credibility and illustrating the extent of human interference with our planet. Finally, teaching and communicating the past evolution of the Earth system can help guide our thinking toward a more sustainable future by overcoming two root problems of the sustainability crisis, the perceived disconnect between humans and the environment, and the lack of long‐term thinking.

## Abstract 2

Historical and future climate change in the context of the past 66 million years, highlighting the importance of Earth's history for the sustainability debate.

## Graphical abstract

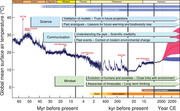

## 1 Introduction

Sustainability is typically defined as a long‐term goal of human societies developing toward a future in which mankind and the environment co‐exist in a way that ensures the well‐being of present and future generations [1]. By definition, the sustainability debate is therefore primarily concerned with the present and future of our planet and our societies. This does not imply, of course, that sustainability considerations are not informed by looking back and learning from past mistakes. On the contrary, the history of environmental problems and ecological crises has been crucial in developing the concept of sustainability. Nevertheless, I would argue that the value of understanding the past is still underestimated – with respect to scientific insights, science communication and its relevance for transforming the human mindset more toward longer‐term thinking. In my opinion, these elements are essential for the sustainability debate, and Earth's past urgently needs more attention in terms of funding, research, science communication, and education.

**FIGURE 1:**
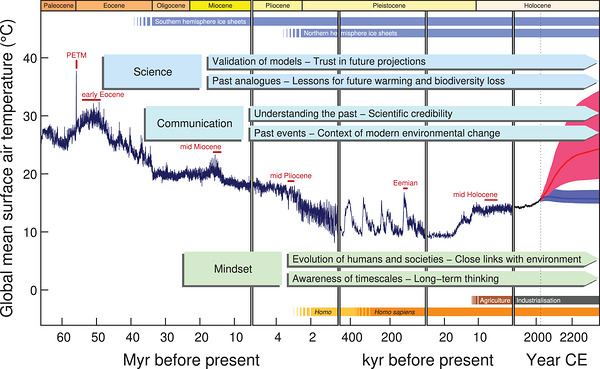
Climate change over the last 66 million years, into the future, and relevance of understanding the past. Illustrative global mean temperature curve based on reconstructions (dark blue) [18, 19, 20], instrumental measurements (black) [21], and future projections until the year 2350 for business‐as‐usual (red) and climate‐mitigation (blue) scenarios [5]. Past warm periods are indicated in red (PETM – Paleocene–Eoecene Thermal Maximum). The glaciation histories of the Southern and Northern hemisphere are indicated toward the top, key milestones in the evolution of the human species and societies at the bottom of the figure.

Looking at my own field, climate science, investigating Earth's climate history might not seem to deserve highest priority given the fundamental environmental and other challenges humanity is facing in the 21st century [2, 3, 4]. However, this view would be as short‐sighted as the predominant attitude toward the sustainability problem. In fact, the value of studying past warm climate states, for example, has long been recognized, in particular in terms of testing numerical models used for projecting future climate change, and of comparing observed current and expected future warming to past analogue states. I would argue, however, that the value of Earth's past for the sustainability debate is not limited to these (albeit important) avenues. Beyond any doubt, there is an inherent cultural importance attached to all questions relating to our origins, of course. But understanding and communicating Earth's history can also enhance credibility when talking about future risks, put the current planetary crisis into context, demonstrate the close connection of humans and human societies with the natural environment, and foster long‐term thinking (see Figure 1). These are essential points for communicating environmental challenges both to the general public and decision makers – and thus prerequisites for building a sustainable future.

## 2 Earth's Past is Important to Better Understand and Model the Earth System

### 2.1 The Past Allows Validation of Climate Models for Future Projections

Let's begin with the importance of the past for Earth system science. One of the key concerns with respect to sustainability is climate change and how it will continue depending on humanity's past and future greenhouse‐gas emissions. Therefore, projections of possible future climate states based on emissions calculated from socio‐economic scenarios are a key element of the regular assessment reports by the Intergovernmental Panel on Climate Change (IPCC) [5]. These insights into our future in a warming world are essential for quantifying the risks of continued climate change and for planning appropriate adaptation measures. However, the Earth‐system models used for future projections are typically tuned and validated against present‐day observations, which does not necessarily imply that they are well suited for the warmer climate states expected in the future. It has long been recognised that one very valuable and effective approach to overcome this limitation is to apply these models to past climates and validate them against reconstructions [6], although the latter inevitably carry uncertainties. Because of the value of past climate states for model validation, the Paleoclimate Model Intercomparison Project (PMIP) has become a well‐established part of the Coupled Model Intercomparison Project (CMIP) [7], which forms the basis for the IPCC's assessments. Past climate states thus form an essential testbed for models used for future projections, thus using the past to build trust in our ability to project future changes.

### 2.2 Past Analogues Provide Insights into a Warmer Future

Another way of assessing our future in a warming world is to investigate warm periods in the past. Earth has experienced a number of warmer climate states in its history (see Figure 1). Due to a variety of changes in the Earth system over time, most prominently maybe the distribution of landmasses on the globe and the evolution of the biosphere, these past warm states will never be perfect analogues for the warming we observe now or will face in the future, even setting aside the almost unprecedented rate of current warming. Nevertheless, a comparison is possible in principle, and depending on time and future emissions, Earth will experience climate states broadly similar to the mid‐Pliocene (about 3 million years ago), the mid‐Miocene (about 15 million years ago) or the early Eocene (about 50 million years ago) [8, 9]. Moreover, these past warm states can also help constrain the Earth's climate sensitivity (the warming associated with a doubling of carbon dioxide levels compared to pre‐industrial times) to enhanced levels of greenhouse gases (by comparing estimates of past carbon‐dioxide concentrations and temperatures) and the stability of important Earth‐system components like the large ice sheets (by investigating their growth and melting in the past) [10, 11, 12]. One concern, however, could be that the current rate of warming is faster than most climate transitions in the past, potentially limiting the insights to be gained from past warm states. Nevertheless, past analogues provide essential information guiding climate policy and thus steering us toward a more sustainable future. Similarly, past mass extinction events, often associated with perturbations of Earth's carbon cycle similar in magnitude to the one resulting from anthropogenic carbon emissions [13], can teach us about the impacts of modern climate change on the biosphere.

While the relevance of past warm climate states and mass extinctions for model testing and for assessing our future in a warmer world is generally acknowledged, funding for paleoclimate and paleobiology research should not be limited to these topics. In fact, the relevance of fundamental research into Earth's history for understanding interactions and feedbacks in the Earth system – even beyond the topic of past warm climate states and biodiversity crises – is frequently neglected.

## 3 Earth's Past is Important for Communicating the Sustainability Challenge

### 3.1 Understanding the Past Enhances Scientific Credibility

Beyond the importance of the past from a scientific perspective, the past is highly relevant for communication to the public and stakeholders. In my experience from many years of giving public lectures on the climate and biodiversity crisis, the mere fact that I do research on Earth's past and can explain why the climate has changed and why we had extinction events in the past significantly enhances my credibility with the general public when warning of the dangers of future warming and increasing biodiversity loss. This goes way beyond countering the well‐known (and spurious) “but the climate has always been changing” argument, or the validation of Earth‐system models discussed below. Even people not prone to denialism (rightfully) demand some sort of reassurance that scientists know what they are talking about. Understanding and communicating past changes in the Earth system is a key element for building credibility in this context. It also taps into the public curiosity about the origins of our planet and our species.

### 3.2 Past Events are Useful to Put Modern Environmental Change Into Perspective

Past climate variations and biodiversity crises can also help illustrate the degree to which humanity has been altering the Earth system – and the even more profound future changes. Many people are aware of the pronounced climatic swings between cold and warm periods in the Pleistocene (about 2.6 million to 12 thousand years ago, see Figure 1), for example, and the fact that the next ice age will be delayed by tens of thousands of years due to human greenhouse‐gas emissions is a powerful demonstration of our influence on the Earth system [14]. Equally thought‐provoking, the warming potential of burning vast amounts of coal over decades or centuries is excellently illustrated by the substantial global cooling when that very same coal was formed over millions of years [15]. In a similar manner, past mass extinction events [16], in particular the prominent ‘Big Five’, serve as an important reference point for the modern biodiversity crisis. Although the notion of the ‘Sixth Extinction’ is sometimes debated, modern biodiversity loss (at least on land) appears to be on track to rival the five biggest mass extinction events in Earth's history [17], providing a dramatic illustration of the scale of the problem.

Comparing modern change to past events and their (sometimes catastrophic) consequences makes people aware of the extent of human interference with the Earth system. Looking into the past can thus help along the sustainability transformation both by illustrating the dimension of the problem and the fact that humans have the power to change things for the better – even on the planetary scale. This point is also closely connected to the concept of the Anthropocene [22]: Although the suggested formal definition of the Anthropocene as a geological epoch was rejected in 2024, it cannot be denied that human activities have lead to Earth‐system changes on a planetary scale and that the idea of the Anthropocene has had a considerable impact on both the scientific and public debate [23, 24, 25, 26].

Despite the high relevance of past Earth system changes for communication, this aspect is often neglected. In my opinion, this constitutes a missed opportunity in terms of building trust and communicating the urgency of the current environmental crisis both to the general public and to stakeholders, potentially contributing to delays in decisive action on the path toward a sustainable future.

## 4 Earth's Past is Important for Fostering Sustainable Thinking

### 4.1 Evolution of Humans and Societies Demonstrates Our Connection to the Natural Environment

Understanding and communicating the co‐evolution of Earth and humans and their societies can also help remove two obstacles on the path toward a sustainable future deeply engrained in our thinking. The first of these obstacles is a perceived disconnect between humans and the natural environment, one of the byproducts of the industrial revolution and the technological advances associated with it. This sense of disconnect is often regarded as one of the root problems of the sustainability crisis [27]. Paleo‐evidence shows, however, that both the evolution and the dispersal of humans [28] as well as the evolution of human societies [29] are intricately linked to environmental changes, also providing a warning with respect to the risks of the current ecological crisis. Making people understand that modern humans and their societies have co‐evolved with the natural environment and depend on it in a myriad of ways can help overcome this disconnect and lead to a more sustainable co‐existence with nature, provided that these insights reach the public consciousness through communication and education.

### 4.2 Awareness of Timescales Helps Foster Long‐Term Thinking

It can be convincingly argued that the second major obstacle on our path toward a sustainable future is the fact that we tend to be stuck in short‐term thinking. This is particularly obvious for our economic and political systems, which are focussed on the cycles of financial reporting and elections, respectively, rather than the long‐term perspective. In her book “Timefulness” [30], Marcia Bjornerud argues that an improved awareness of the timescales over which our planet and its biosphere have co‐evolved could help overcome this problem, although the communication of timescales can be challenging [31]. I share the view that awareness of past timescales can foster long‐term thinking but this requires an emphasis on these topics in the curriculum, which are often not covered at all, and increased efforts in science communication from the paleo‐science community. Awareness of long timescales is also particularly relevant in light of the fact that our actions today can have long‐term legacy effects on the future Earth system, for example in terms of sea‐level rise [32].

The scientific discipline which was instrumental in deciphering the timescales of Earth's history is geology, of course, and geology has a key role in providing the foundations of the long‐term perspective required for sustainability. At the same time, geology is under significant pressure because of its role in fossil‐fuel exploration and extraction, potentially jeopardising its contribution to the sustainability debate. In addition to exploration of resources required for the energy transition, demonstrating its value in terms of longer‐term thinking might help geology as a discipline; in addition, other fields of Earth science need to put more emphasis on this longer‐term perspective on our planet.

The perceived disconnect between us and the environment and our inability to think ahead on longer timescales, both exacerbated by the technologies of the digital era, can certainly be regarded as major obstacles on our path into a sustainable future. Raising awareness of our co‐evolution with the natural environment and of timescales in themselves will not be sufficient to overcome these, of course, but communication of Earth's past should be an important element.

## 5 Conclusions

The arguments presented above demonstrate the value of investigating Earth's past and communicating and teaching the findings. In my opinion, this is not sufficiently recognised by funding agencies, the scientific community, science communicators, and the educational sector. In terms of funding, the value of research in fields like paleoclimatology and paleobiology for sustainability should be recognised. Even if insights do not directly contribute to sustainability policies, they can still improve our understanding of how the Earth system works. Furthermore, communication on the dimension of the current environmental crisis benefits from taking past changes into account, both in terms of enhancing scientific credibility and illustrating the extent of human interference with our planet. Finally, school and university curricula should put more emphasis on the past evolution of the Earth system, and in particular the co‐evolution of humans and their societies with the environment to overcome two root problems of the sustainability crisis, the perceived disconnect between humans and the environment and a lack of longer‐term thinking.

One way to accomplish the goals outlined above is by tapping into the native curiosity of human beings about the origins of our universe, our planet, our species, and our societies. Furthermore, storytelling has proven to be a powerful tool to draw attention to these topics on all levels, as evidenced by the scientific and public interest in narratives about the formation of Earth [33], the origin of life [34], planetary habitability [35, 36], Snowball Earth events [37], the evolution [38] and demise [39, 40] of charismatic animals like the dinosaurs, and the interplay between past climate change and the evolution of humans [28] and their societies [29].

In summary, research into and communication of Earth's history is highly relevant in the context of the climate change, biodiversity, and the broader sustainability debates. This importance is often overlooked, however, and needs to be recognized by funding agencies, the scientific community, and the educational sector alike. A core concept of modern geology states that “the present is the key to the past”. I would argue that the past is also the key to the future.

## Conflicts of Interest

The author declares no conflicts of interest.

## Data Availability

No new data were created for this research. Data plotted in Figure 1 were obtained from existing public repositories: [41, 42, 43, 44, 45, 46].
